# Development of an electronic health record-based chronic kidney disease registry to promote population health management

**DOI:** 10.1186/s12882-019-1260-y

**Published:** 2019-03-01

**Authors:** Mallika L. Mendu, Salman Ahmed, Jason K. Maron, Sandhya K. Rao, Sreekanth K. Chaguturu, Megan F. May, Walter P. Mutter, Kelly A. Burdge, David J. R. Steele, David B. Mount, Sushrut S. Waikar, Jeffrey B. Weilburg, Thomas D. Sequist

**Affiliations:** 1 Division of Renal Medicine, Brigham and Women’s Hospital, Harvard Medical School, One Brigham Circle, Boston, MA 02115 USA; 2 Partners Healthcare, Partners eCare, Boston, MA USA; 3 Partners Healthcare, Center for Population Health Management, Boston, MA USA; 4 Division of Nephrology, Newton Wellesley Hospital, Boston, MA USA; 5 Division of Renal Medicine, North Shore Medical Center, Boston, MA USA; 6 Division of Renal Medicine, Massachusetts General Hospital, Harvard Medical School, Boston, MA USA; 7 Partners Healthcare, Quality Safety and Value, Boston, MA USA; 8 Division of General Medicine, Brigham and Women’s Hospital, Boston, MA USA; 9 Department of Health Care Policy, Harvard Medical School, Boston, MA USA

**Keywords:** Chronic kidney disease, Population health management, Registry, Electronic health record

## Abstract

**Background:**

Electronic health record (EHR) based chronic kidney disease (CKD) registries are central to population health strategies to improve CKD care. In 2015, Partners Healthcare System (PHS), encompassing multiple academic and community hospitals and outpatient care facilities in Massachusetts, developed an EHR-based CKD registry to identify opportunities for quality improvement, defined as improvement on both process measures and outcomes measures associated with clinical care.

**Methods:**

Patients are included in the registry based on the following criteria: 1) two estimated glomerular filtration rate (eGFR) results < 60 ml/min/1.73m^2^ separated by 90 days, including the most recent eGFR being < 60 ml/min/1.73m^2^; or 2) the most recent two urine protein values > 300 mg protein/g creatinine on either urine total protein/creatinine ratio or urine albumin/creatinine ratio; or 3) an EHR problem list diagnosis of end stage renal disease (ESRD). The registry categorizes patients by CKD stage and includes rates of annual testing for eGFR and proteinuria, blood pressure control, use of angiotensin converting enzyme inhibitors (ACE-Is) or angiotensin receptor blockers (ARBs), nephrotoxic medication use, hepatitis B virus (HBV) immunization, vascular access placement, transplant status, CKD progression risk; number of outpatient nephrology visits, and hospitalizations.

**Results:**

The CKD registry includes 60,503 patients and has revealed several opportunities for care improvement including 1) annual proteinuria testing performed for 17% (stage 3) and 31% (stage 4) of patients; 2) ACE-I/ARB used in 41% (stage 3) and 46% (stage 4) of patients; 3) nephrotoxic medications used among 23% of stage 4 patients; and 4) 89% of stage 4 patients lack HBV immunity. For advanced CKD patients there are opportunities to improve vascular access placement, transplant referrals and outpatient nephrology contact.

**Conclusions:**

A CKD registry can identify modifiable care gaps across the spectrum of CKD care and enable population health strategy implementation. No linkage to Social Security Death Master File or US Renal Data System (USRDS) databases limits our ability to track mortality and progression to ESRD.

**Electronic supplementary material:**

The online version of this article (10.1186/s12882-019-1260-y) contains supplementary material, which is available to authorized users.

## Background

Chronic kidney disease (CKD) is a major public health problem that affects over 25 million adults in the United States [1]. Patients with CKD have a significant risk of progressing to end stage renal disease (ESRD) and requiring either dialysis or kidney transplantation [2–5]. Unfortunately, as patients progress they experience an increased risk of death, from 20% for Stage 3 CKD to 300% for stages 4 and 5 CKD, when compared to individuals with eGFR ≥60 ml/min, which may include stage 1 and 2 CKD patients [4]. This clinical burden of CKD is accompanied by a substantial financial burden, generating costs of $23 billion for ESRD management and nearly $50 billion for non-ESRD related CKD management [6].

Given the significant burdens associated with CKD, it is imperative to develop population health initiatives to address CKD care [7]. Clinical registries, particularly those based on electronic health records (EHR), form the foundation of such population-based improvement activities. These registries facilitate identification of patients, capture clinical quality metrics, and track clinical outcomes [8–11]. Registries coupled with care management yield significant improvements in clinical outcomes [8, 9].Compared to other less prevalent chronic conditions, there has been limited investigation regarding the development and implementation of CKD registries [12–19].

In 2015, PHS adopted a network-wide EHR (Epic Systems, Verona, WI) and implemented a CKD registry. The goals of the CKD registry included: 1) identification of patients with CKD based on laboratory data (eGFR and proteinuria) 2) demonstration of performance on quality metrics and care delivery gaps; and 3) tracking of clinical outcomes such as CKD progression and health care utilization. In this manuscript, we describe our process of developing a CKD registry, describe our performance on quality metrics, and outline the implementation of population health strategies to address gaps in care.

## Methods

### Setting

PHS is a non-profit hospital and provider network based in eastern Massachusetts, serving close to 6 million patients. Brigham and Women’s Hospital (BWH) and Massachusetts General Hospital (MGH) are the flagship tertiary referral academic centers in the organization, with highly specialized medical and surgical services offered. Several traditional community medical centers provide general inpatient services. Several outpatient practices spread throughout the greater Boston area are part of PHS. Starting in 2015, a common EHR has been used by ambulatory and inpatient providers across the network. We organized a system-wide CKD collaborative, comprised of nephrologists and Primary Care Providers (PCPs). This collaborative has provided oversight of the registry development.

### Patient identification for inclusion in CKD registry

**Fig. 1:**
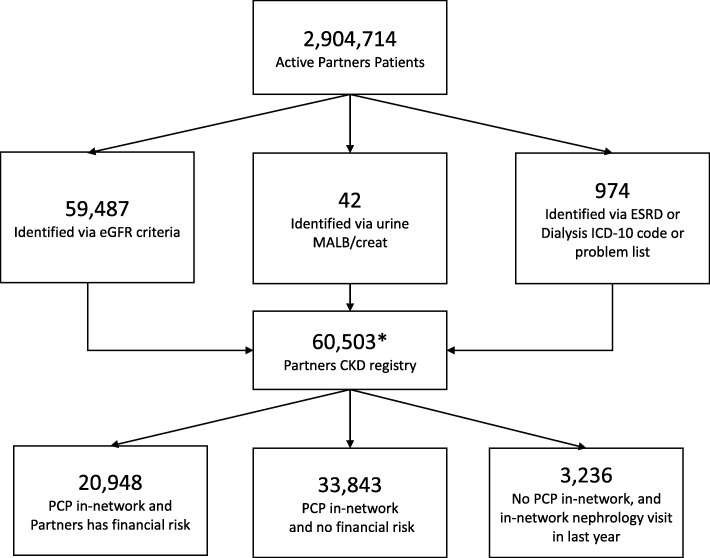
Identification of Partners HealthCare System patients for inclusion into the CKD Registry Abbreviations: eGFR - estimated glomerular filtration rate, urine MALB/creat - urine albumin to creatinine ratio, urine prot/creat - urine protein to creatinine ratio, ESRD - End Stage Renal Disease. *2476 patients received care from a non-nephrologist specialist and therefore did not have either a Partners PCP or Partners Nephrologist

Figure 1 depicts inclusion criteria for the CKD registry, including laboratory data, diagnosis codes, and visit data. Patients are included in the CKD registry first by identifying alive patients at least 18 years old that are ‘active’ in our system, defined as having a prior ambulatory or inpatient encounter within the previous 5.5 years or a future scheduled encounter; as well as having recently updated wellness information such as vaccinations or laboratory values.

Among these active adult patients, we include patients if they meet one of the following criteria: 1) most recent eGFR < 60 ml/min/1.73m^2^ and one additional eGFR at least 90 days prior < 60 ml/min/1.73m^2^, with both values recorded within the last 3 years;or 2) at least two values of urine total protein or urine albumin > 300 mg/gm; or 3) ESRD or dialysis on EHR problem list or as an International Classification of Diseases (ICD)-10 diagnostic code during an ambulatory or inpatient encounter. Prior to March 2018, the EHR calculated eGFR using the Modification of Diet in Renal Disease (MDRD) equation. Subsequently, the EHR has utilized the Chronic Kidney Disease Epidemiology Collaboration (CKD-EPI) serum creatinine equation, adjusting for African American race by multiplying generic eGFR values by 1.212.

Our CKD registry includes 60,503 patients as of July 31, 2018. The registry updates data in real-time, so the total number of patients included varies daily. The vast majority of patients (98%) are identified via eGFR values, and 91% have a PCP within Partners (Fig. 1).

### CKD and 5D staging

**Fig. 2:**
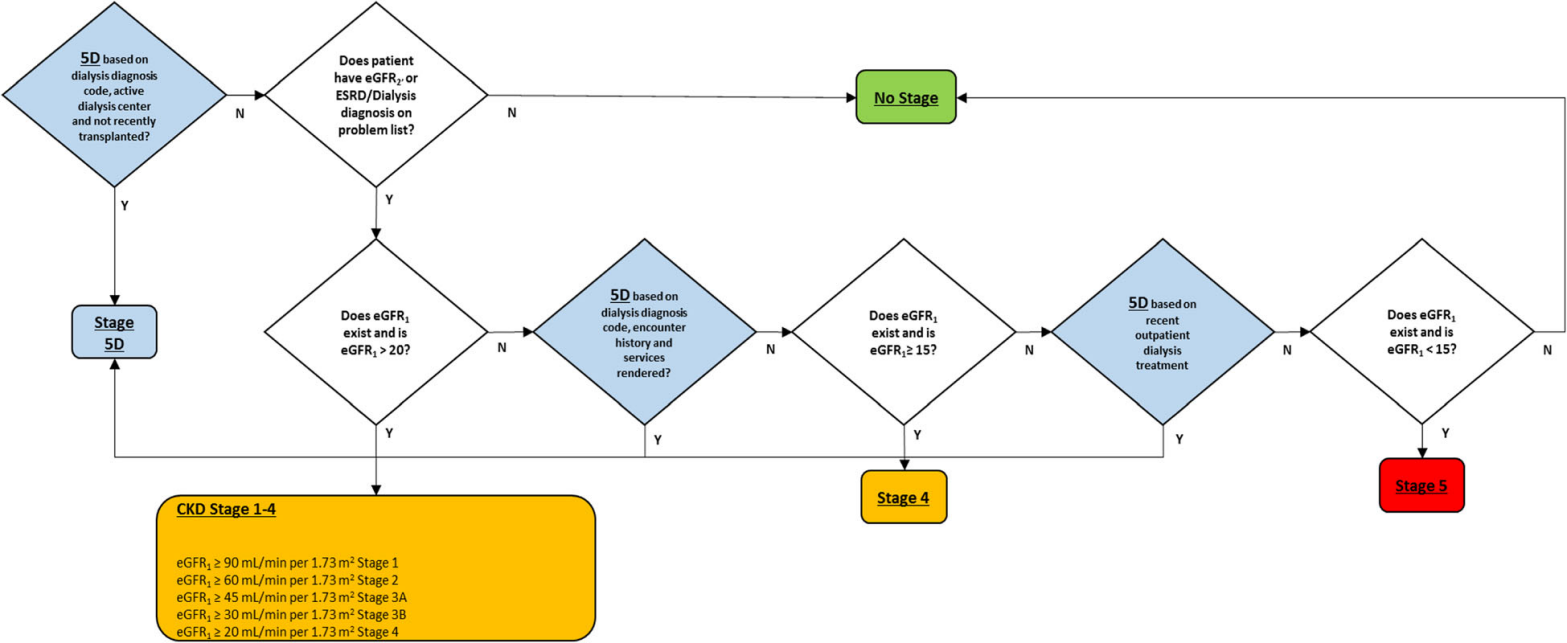
Staging algorithm employed in PHS CKD Registry Abbreviations: ESRD- End Stage Renal Disease; eGFR- estimated glomerular filtration rate. Definitions: eGFR_1_ = The most recent eGFR value within the last 3 years, eGFR_2_ = The second most recent eGFR value within the last 3 years, eGFR_2_’ = The most recent eGFR value within the last 3 years that occurred at least 90 days prior to eGFR_1_

Our CKD collaborative developed the algorithm for classifying patients in stages 1 through 5 (Fig. 2). Given that eGFR can fluctuate for a patient over the course of several years, there are multiple approaches to such classification, including ‘average eGFR’, ‘lowest eGFR’, and ‘most recent eGFR’. We opted to base classification on the most recent eGFR to reduce confusion among front line clinicians, recognizing the limitation that this may sometimes capture acute kidney injury.

We classified patients as 5D (requiring dialysis) or ESRD based on the presence of active dialysis ICD-9 or ICD-10 codes (including such common codes as ICD-9 CM: V56.0, V56.1, 585.6, V45.11; and ICD-10 CM: N18.6, Z99.2) or recent outpatient dialysis treatments regardless of eGFR (see Additional file 1: Table S1). We classified patients as stage 5 if no criteria for active dialysis is met and the most recent eGFR is < 15. Patients are classified as ‘no stage assigned’ if there are no eGFR values in the last 3 years and no active dialysis criteria are met.

### CKD registry metrics

The CKD registry metrics include demographics, labs, immunizations, medications, procedural history (vascular access), transplant status, blood pressure, 2-year and 5-year Tangri progression risk score [20] and healthcare utilization (Table 1). These data are displayed for clinicians in a user-friendly format that shows opportunities for improvement at a glance (Additional file 2: Figure S1). Our quality metrics were chosen based on published guidelines [21] as well as consensus from CKD collaborative members on standard CKD management. The medications included as unsafe resulted from PHS CKD collaborative meetings (Additional file 3: Table S2), and include metformin, bisphosphates, and nitrofurantoin [22]. The goal is to signal to the provider that a patient could be on a medication that needs to be discontinued or dose adjusted.

**Table 1:** Data and metrics included in the chronic kidney disease registry

	Definition (if applicable)	Categorization (if applicable)
Patient Characteristics		
Age		
Sex		
Race		White; African-American; Hispanic; Other
Comorbidities		Diabetes; HTN; CHF; CVD; COPD; Asthma; Obesity
Insurance		Medicare; Medicaid; Commercial; Other; Unknown
PCP		Name, last date of visit
Nephrologist		Name, last date of visit
Disease Characteristics		
CKD stage		Stages 3A, 3B, 4, 5, 5D
Kidney failure risk score	2- and 5- year risk of progression to ESRD^a^	2-year, 5-year risk of progression
Rate of eGFR change	% change in eGFR by at least 90 days	
Laboratory Data		
Serum creatinine		Continuous by mg/dl
eGFR^b^		Continuous by ml/min/1.73 m2
Metrics		
ED Visits, n/per year		
Inpatient Visits, n/per year		
Outpatient PCP Visits, n/per year		
Outpatient Nephrology Visits, n/per year		
Annual creatinine		Yes or no, date obtained, value
Annual urine protein testing		Yes or no, date obtained, value
Blood pressure control		Yes, no, or unknown
ACE-I/ARB use		Yes, no, or not applicable^c^
Nephrotoxin status	Review of patient electronic medication list for presence of any one of several medications thought to be unsafe in patients with CKD, based on KDIGO guidelines and CKD collaborative consensus	1. Prescribed no renally unsafe medications 2. Prescribed potentially unsafe medications 3. Prescribed renally unsafe medication (eGFR ≤30)
Hepatitis B immunity status		1. Immune by HbsAb 2. Immunization received 3. Not immune by HBsAb
Patient reported outcome measures (PROMs)		Completed, not completed, scores
Placement of AVF/AVG^d^		Yes, no
Transplant status^e^		1. None 2. Referral 3. Evaluation 4. Waitlist 5. Transplanted

*Abbreviations*: *EHR* Electronic health record, *HTN* Hypertension, *CHF* Congestive heart failure, *CVD* Cardiovascular disease, including coronary artery disease, peripheral vascular disease and stroke, *COPD* Chronic obstructive pulmonary disease, *PCP* Primary care provider, *ACE-I* Angiotensin converting enzyme inhibitor, *ARB* Angiotensin receptor blocker, *AVF* Arteriovenous fistula, *AVG* Arteriovenous graft

^a^Based on Tangri et al. Multinational Assessment of Accuracy of Equations for Predicting Risk of Kidney Failure: A Meta-analysis. JAMA 2016

Patients included based on presence of eGFR and urine protein quantification separated by less than or equal to 1 year (total of 5487 patients)

^b^Prior to March 2018, the EHR calculated eGFR using the Modification of Diet in Renal Disease (MDRD) equation; subsequently, the EHR has utilized the Chronic Kidney Disease Epidemiology Collaboration (CKD-EPI) serum creatinine equation; adjusted for African American race by multiplying generic eGFR values by 1.212

^c^“not applicable” if patient did not have hypertension, diabetes, or proteinuria, or if they did have last serum potassium > 5.0 meq/L, bilateral renal artery stenosis, documented nephrotoxic reaction to ACE-I/ARB, documented angioedema or swelling reaction to ACE-I/ARB, or were pregnant

^d^Presence of a CPT code, ICD-10 or dialysis access documentation field within EHR for AVF and/or AVG

^e^Presence of “transplant status” field within EHR

### Data management

The CKD registry is available for use by individual clinicians managing their own patient panel as well as clinical directors overseeing an entire CKD population. The data collected within the EHR registry are backed up nightly to a separate enterprise wide data warehouse, where the data can be combined with external data sources such as claims and cost data to facilitate more advanced analytics. Only approved users within the PHS network who have been trained on appropriate use and stewardship of data are permitted to access and query the EDW to obtain aggregate data across providers and clinics.

### Registry data validation

We validated the registry data using two sequential steps. First, 100 patients were randomly selected from each of stage 4, 5, and 5D designations as determined by our CKD staging algorithm (Fig. 1). Two study authors (SA and MFM) performed chart reviews to assess for appropriate staging, the presence or absence of arteriovenous fistula or arteriovenous graft (AVF or AVG), and transplant status. This first step allowed refinement of our CKD staging algorithm for 5D patients, such as adding logic to capture inpatient dialysis progress notes.

Second, we randomly identified an additional 300 patients with stage 4, 5, and 5D CKD for additional manual chart review. We performed additional validation of stage 3 patients. We demonstrated a positive predictive value (PPV) of 100% (95% CI 96.4–100%) for stage 3 and 4 patients, 82% (95% CI 73.1–89.0%) for stage 5 patients, and 91% (95% CI 83.6–95.8%) for stage 5D patients. Nearly all (99%) patients who were classified as stage 5 or 5D were validated as having at least stage 5. However, we found that patients were sometimes classified as stage 5D when not on dialysis, or stage 5 when on dialysis, (11 and 20% of cases, respectively). This could be due to documentation of dialysis treatment only in scanned documents not identifiable in automated data extracts, recent dialysis discontinuation mentioned in free text notes, or reversal of decisions to pursue dialysis.

Our second chart review also demonstrated a PPV of 100, 91 and 83% for vascular access for stages 4, 5, and 5D respectively; and a PPV of 93, 95 and 79% for transplant status. Most discrepancies were due to information contained within the body of progress notes.

## Results

### Patient demographics and clinical characteristics

We identified 60,503 patients with CKD, with the majority (84%) having stage 3 CKD, and the remainder having Stage 4 (9%), Stage 5 (2%), or Stage 5D (5%) CKD (Table 2). The average patient was above 60 years for all stages, and African American and Hispanic patients were disproportionately represented among Stages 5 and 5D. Our patients with CKD experience a high burden of hypertension, diabetes and cardiovascular disease. Among 5487 patients eligible for calculation of Tangri risk score (presence of eGFR and urine protein quantification separated by less than or equal to 1 year), a significant number of stage 4 patients have greater than a 30% risk of progression to ESRD in two and 5 years (12.7 and 39.6%, respectively).

**Table 2:** Characteristics of patients in registry by stage

N%	CKD Stage					
	3A^d^	3B	4	5	5D	Aggregate
Total	32,962 (54.5)	17,927 (29.6)	5579 (9.2)	1295 (2.1)	2740 (4.5)	60,503
Age, years (+/− S.D.)	73.4 (12.1)	77.3 (12.1)	76.5 (13.6)	69.1 (15.9)	63.6 (14.9)	
*≥80 (%)*	*32.3*	*47.1*	*46. 9*	*28.7*	*14.0*	
Female Sex	18,917 (57.4)	10,483 (58.5)	3010 (54.0)	617 (47.6)	1153 (42.1)	34,180 (56.5)
Ethnicity						
White	28,297 (85.9)	15,322 (85.5)	4601 (82.4)	935 (72.2)	1539 (56.2)	50,694 (83.8)
African-American	1066 (3.2)	709 (4.0)	310 (5.6)	143 (11.0)	590 (21.5)	2818 (4.7)
Hispanic	999 (3.0)	499 (2.8)	184 (3.3)	52 (4.0)	226 (8.25)	1960 (3.2)
Other^a^	2600 (7.9)	1397 (7.8)	484 (8.7)	165 (12.7)	385 (14.1)	5031 (8.3)
Kidney Failure Risk^b^						
2-year risk > 30%	1 (0)	5 (0.2)	106 (12.7)	69 (69.7)	116 (85.3)	
5-year risk > 30%	2 (0)	37 (1.8)	331 (39.6)	96 (97.0)	134 (98.5)	
Comorbidities^c^						
Diabetes	8173 (24.8)	6331 (35.3)	2368 (42.4)	533 (41.2)	1356 (49.5)	18,761 (31.0)
HTN	23,717 (72.0)	14,548 (81.2)	4624 (82.9)	993 (76.7)	2158 (78.8)	46,040 (76.1)
CHF	3988 (12.1)	3968 (22.1)	1733 (31.1)	289 (22.3)	877 (32.0)	10,855 (17.9)
CVD	8723 (26.5)	6406 (35.7)	2156 (38.6)	370 (28.6)	1100 (40.2)	18,755 (31.0)
COPD	2736 (8.3)	1916 (10.7)	683 (12.2)	93 (7.2)	269 (9.8)	5697 (9.4)
Asthma	3472 (10.5)	1757 (9.8)	529 (9.5)	85 (6.6)	247 (9.0)	6090 (10.1)
Obesity	10,054 (30.5)	5446 (30.4)	1676 (30.0)	303 (23.4)	853 (31.1)	18,332 (30.3)
Payor						
Medicare	23,198 (70.4)	14,446 (80.6)	4303 (77.1)	875 (67.6)	1959 (71.4)	
Medicaid	1096 (3.3)	497 (2.8)	206 (3.7)	91 (7.0)	253 (9.2)	
Commercial	4190 (12.7)	1337 (7.5)	438 (7.9)	119 (9.2)	183 (6.7)	
Other/Unknown	4478 (13.6)	1647 (9.2)	632 (11.3)	210 (16.2)	349 (12.7)	

*Abbreviations*: *HTN* Hypertension, *CHF* Congestive heart failure, *CVD* Cardiovascular disease, including coronary artery disease, peripheral vascular disease and stroke, *COPD* Chronic obstructive pulmonary disease

^a^Other includes Asian, Native American, Hawaiian/Pacific Islander, Mixed, and Unknown

^b^Based Tangri et al. Multinational Assessment of Accuracy of Equations for Predicting Risk of Kidney Failure: A Meta-analysis. JAMA 2016. Patients included based on presence of eGFR and urine protein quantification separated by less than or equal to 1 year (total of 5487 patients). Denominators are as follows (3A 2382, 3B 2034, 4836, 5 99, 5D 136)

^c^Comorbidities identified using ICD-10 codes as well as problem list documentation in the EHR

^d^Includes patients with proteinuria of > 300 mg/g creatinine but with normal eGFR

### Assessment of quality metrics

The CKD registry presents data on five key quality metrics (Table 3). Less than one-third of patients with Stages 3 and 4 CKD have received annual testing for proteinuria, and less than one-half of these patients are treated with an ACE-I/ARB. Among patients with stages 4, 5, and 5D CKD, as many as one-quarter (23%) are treated with a potentially unsafe or nephrotoxic medication. A majority of patients with stage 4 (89%) and stage 5 (75%) CKD have no evidence of immunity to hepatitis B.

**Table 3:** Performance on CKD Management Evidence-Based Quality Metrics captured by PHS CKD registry

N (%)	CKD Stage				
	3A (*n* = 32,962)	3B (*n* = 17,927)	4 (*n* = 5579)	5 (*n* = 1295)	5D (*n* = 2740)
Annual Testing ^a^					
eGFR	26,725 (81.1)	14,622 (81.6)	4374 (78.4)	787 (60.8)	*
Proteinuria	5734 (17.4)	4276 (23.9)	1738 (31.2)	240 (18.5)	*
BP control^b^					*
Yes	24,153 (73.3)	12,868 (71.8)	3708 (66.5)	660 (46.8)	*
No	2784 (8.5)	1334 (7.4)	409 (8.7)	112 (8.7)	*
Unknown	6025 (18.3)	3725 (20.8)	1462 (26.2)	577 (44.6)	*
ACE-I/ARB^c^					*
Yes	13,766 (41.8)	8308 (46.3)	2292 (41.1)	409 (31.6)	*
No	10,956 (33.3)	6869 (38.3)	2570 (46.1)	655 (50.6)	*
N/A	8240 (25.0)	2750 (15.3)	717 (12.9)	231 (17.8)	*
Nephrotoxin Status^d^					
Not on any renally unsafe medications	NC	NC	4286 (76.8)	1075 (83.0)	1986 (72.5)
On potentially unsafe medication	NC	NC	929 (16.7)	199 (15.4)	489 (17.9)
On renally unsafe medication (eGFR≤30)	NC	NC	364 (6.5)	21 (1.6)	14 (0.5)
Hepatitis B immunity^e^					
Immune by HBsAB	553 (1.7)	339 (1.9)	178 (3.2)	137 (10.6)	553 (20.2)
Immunization received	1935 (5.9)	924 (5.2)	415 (7.4)	192 (14.8)	876 (32.0)
Not immune by HBsAB	30,474 (92.5)	16,664 (93.0)	4986 (89.4)	966 (74.6)	1311 (47.9)

*Abbreviations*: *eGFR* Estimated glomerular filtration rate, *ACE-I* Angiotensin converting enzyme inhibitor, *ARB* Angiotensin receptor blocker, *HBsAB* Hepatitis B surface antibody

^*^Data not included due to lack of evidence-base for quality metrics in 5D patients

*N/A* Not applicable as defined below

*NC* Not calculated for CKD stage 3a and 3b, given lack of evidence-base for nephrotoxin avoidance in patients with eGFR > 30

Definitions:

^a^Annual Testing indicates the proportion of patients who had at least one recorded value for each designated test within the past year

^b^Blood pressure control: Patients who had the most recent recorded blood pressure < 140/90 mmHg or on maximal blood pressure medication therapy (defined as three anti-hypertensive agents at maximum dose, including diuretics were designated as “yes.” Those with no recorded blood pressure values at all or those with no blood pressure values recorded within the last year were designated as “unknown.” All others were designated as “no.”

^c^ACE-I/ARB: Patients were designated as “N/A”, not applicable, if they did not have hypertension, diabetes, or proteinuria, or if they did have last serum potassium > 5.0 meq/L, bilateral renal artery stenosis, documented nephrotoxic reaction to ACE-I/ARB, documented angioedema or swelling reaction to ACE-I/ARB, or were pregnant. Patients who were prescribed ACE-I or ARB were designated as “yes.” All other patients were designated as “no.”

^d^Nephrotoxic medication list was derived based on KDIGO 2012 guidelines,^22^ Whittaker et al. CJASN 2018^23^ (initially preliminary data shared by this research group). “Renally unsafe medications” are medications that are contraindicated in patients with eGFR< 30 ml/min/1.73m^2^. “Potentially unsafe medications” are medications that may result in toxicity in those patients with <eGFR. Please see Additional file 3: Table S2 for the complete lists of both types of medications

^e^Hepatitis B immunity is defined as having received a HBV vaccine or having a HBsAB titer > 12 mIU/ml. If at least one of these conditions was not met, then patients were categorized as “Not immune by HBsAB.”

### Vascular access placement

Two-thirds (62.0%) of patients with stage 5D CKD have undergone placement of either an AVF or AVG (Table 4), while one-quarter (22%) of stage 5 CKD patients have undergone placement of either AVF or AVG.

**Table 4:** Rates of vascular access placement and transplant evaluation

N (%)	CKD Stage		
	4 (*n* = 5579)	5 (*n* = 1295)	5D (*n* = 2740)
Placement of AVF or AVG^a^	158 (2.8)	290 (22.4)	1700 (62.0)
Transplant Status^b^			
None	5095 (91.3)	819 (63.2)	1290 (47.1)
Referral	74 (1.3)	73 (5.6)	239 (8.7)
Evaluation	92 (1.7)	136 (10.5)	437 (16.0)
Waitlist	161 (2.9)	227 (17.5)	590 (21.5)
Transplanted	157 (2.8)	40 (3.1)	184 (6.7)

*Abbreviations*: *AVF* Arteriovenous fistula, *AVG* Arteriovenous graft

Definitions:

None- patient has not been referred for transplant

Transplanted- indicates that the patient has previously undergone renal transplant and is categorized into a CKD stage based on current, post-transplant eGFR

^a^Determined by presence of a CPT code, ICD-10 or dialysis access documentation field within EHR for AVF and/or AVG

^b^Determined by presence of “transplant status” field within EHR

### Transplantation status

Slightly more than one-half (54%) of all stage 5D patients have been referred for transplantation (Table 4), and 37% of stage 5 patients have been referred. A small proportion (2%) of patients representing each CKD stage are prior transplant recipients.

### Utilization metrics

A minority (14%) of patients with stage 4 CKD have had an office visit with nephrology three or more times in the past year (Table 5). A visit to a nephrologist was defined as a single ambulatory encounter with a nephrologist. It was assumed that nephrologist visits by patients in this CKD registry were related to CKD as a primary or secondary visit diagnosis. 45% of ESRD patients present to the emergency department (ED) at least once per year, and 47% are admitted to the hospital at least once per year. Frequency of inpatient visits increase with increasing CKD severity, except among patients with stage 5 CKD (non-dialysis).

**Table 5:** Outpatient and Inpatient visits per year, by CKD Stage

N %	CKD Stage				
	3A (*n* = 32,962)	3B (*n* = 17,927)	4 (*n* = 5579)	5 (*n* = 1295)	5D (*n* = 2740)
Outpatient Nephrology					
0	30,277 (91.9)	14,789 (82.5)	3722 (66.7)	895 (69.1)	1819 (66.4)
1	1205 (3.7)	1134 (6.3)	424 (7.6)	116 (9.0)	314 (11.5)
2	889 (2.70)	1196 (6.7)	635 (11.4)	58 (4.5)	202 (7.4)
3+	591 (1.79)	808 (4.5)	798 (14.3)	226 (17.5)	405 (14.8)
ED					
0	26,494 (80.4)	13,463 (75.1)	3928 (70.4)	1005 (77.6)	1495 (54.6)
1	3895 (11.8)	2390 (13.3)	765 (13.7)	125 (9.7)	457 (16.7)
2	1366 (4.1)	1008 (5.6)	414 (7.4)	78 (6.0)	270 (9.9)
3+	1207 (3.7)	1066 (6.0)	472 (8.5)	87 (6.7)	518 (18.9)
Inpatient admissions					
0	28,330 (86.0)	14,252 (79.5)	4143 (74.3)	1048 (80.9)	1429 (52.2)
1	3032 (9.2)	2200 (12.3)	759 (13.6)	122 (9.4)	587 (21.4)
2	974 (3.0)	778 (4.3)	337 (6.0)	64 (4.9)	285 (10.4
3+	626 (1.9)	697 (3.9)	340 (6.1)	61 (4.7)	439 (16.0)
30-day Readmissions					
0	32,200 (97.7)	17,192 (95.9)	5237 (93.9)	1225 (94.6)	2326 (84.9)
1	554 (1.7)	513 (2.9)	231 (4.1)	45 (3.5)	232 (8.5)
2	128 (0.4)	133 (0.7)	67 (1.2)	14 (1.1)	92 (3.4)
3+	80 (0.2)	89 (0.5)	44 (0.8)	11 (0.9)	90 (3.3)

*Abbreviations*: *ED* Emergency department visits

## Discussion

We have developed a network-wide CKD registry designed to measure performance on quality metrics and track clinical outcomes over time. We used a continuous process improvement model to validate and refine our registry data based on manual chart review. We demonstrated a level of accuracy for identification of patients and clinical markers that will facilitate robust quality improvement. Our CKD registry has identified population level improvement opportunities amenable to systems interventions.

Few studies have developed and examined the implementation of a CKD registry [4, 12, 15, 17–19]. The Cleveland Clinic developed an EHR-based CKD registry to identify CKD patients based on lab data, and was initially comprised of 65,116 patients [14]. It has been utilized to disseminate CKD stage-specific education, facilitate clinical trials and improve lab monitoring [12, 14, 18]. A recent randomized trial demonstrated that an electronic CKD registry can be used to improve urine protein testing and appropriate nephrology management for stage 3 CKD patients [19]. Finally, a recent trial utilized a CKD registry to facilitate population management strategies including quarterly performance reports to PCPs and point of care management [17].

Our CKD registry is distinct from the registries previously described as it integrates data to drive more comprehensive CKD care improvement. We include information on effectiveness (blood pressure management, use of ACE-I/ARB, and nephrology co-management), safety (nephrotoxic medications and hepatitis B immunization), cost (ED visits and hospitalizations), and clinical outcomes (CKD progression and patient reported outcomes [PROMs]) across the full spectrum of CKD stages.

### Opportunities to create value: the identification of gaps in care

Studies to date illustrate the need for regional or network-based CKD registries that aggregate objective data [23], but have not been focused on clear care gaps that can be utilized by both PCPs and nephrologists. Our CKD registry suggests multiple areas for improvement in the delivery of care. With respect to early CKD management often performed by PCPs, rates of proteinuria testing and ACE-I/ARB treatment should be targeted. For advanced CKD and nephrologist-based care, nephrotoxin avoidance, hepatitis B immunization, vascular access placement and transplant evaluation can be improved. CKD patients in our registry are high utilizers of inpatient care, consistent with findings in the general CKD population [6]. The inclusion of the Tangri risk scores and utilization data will help identify patients at greatest risk and need for intervention.

Within our institution, the first phase of CKD registry implementation will involve education of clinicians across PHS practice sites, followed by implementation of a registry coordinator to promote population health strategies.

### Population health strategies for CKD management

**Fig. 3:**
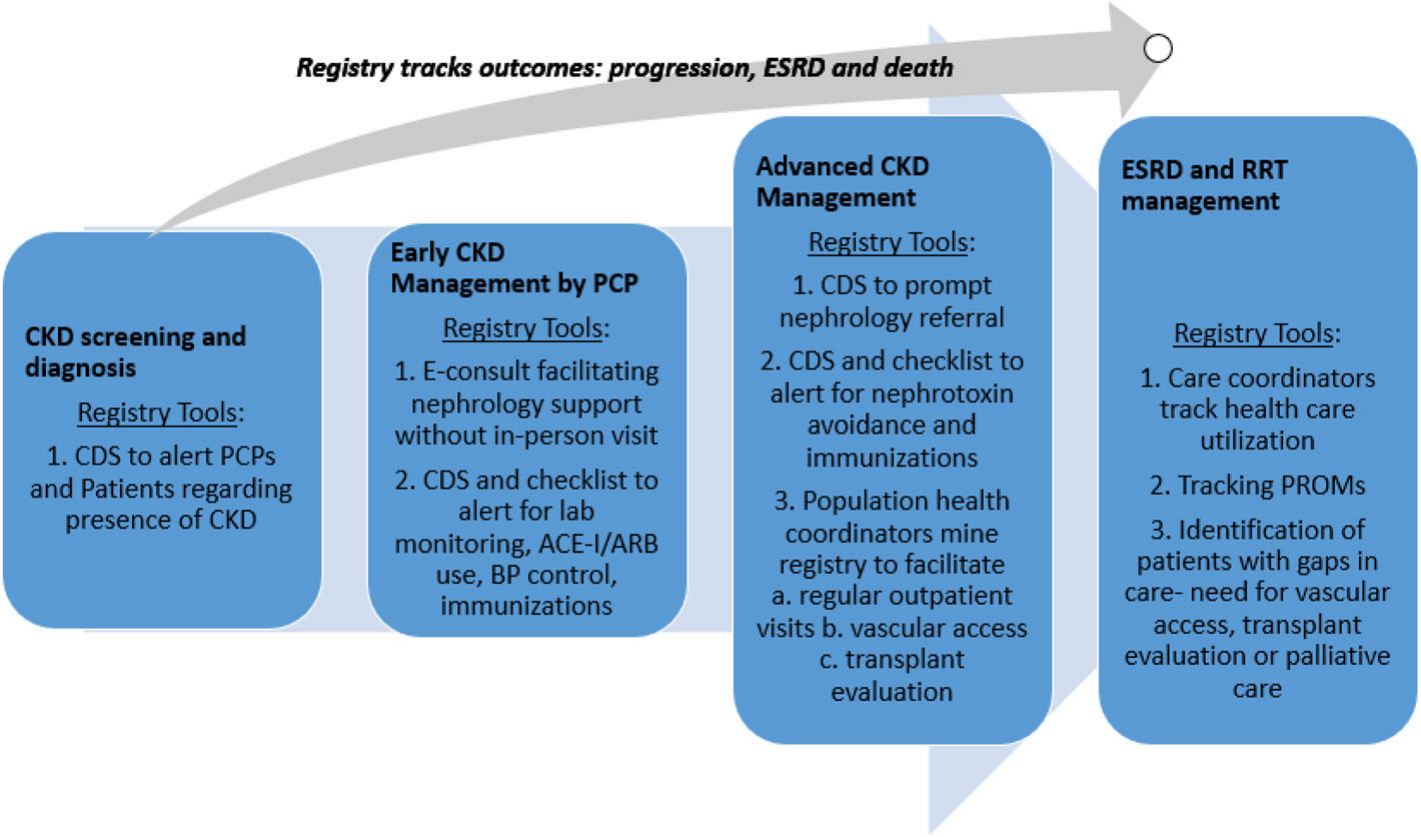
Registry-based tools to facilitate population health strategies across the spectrum of CKD care Abbreviations**:** ESRD- end stage renal disease, CKD- chronic kidney disease, CDS- clinical decision support, PCP- primary care provider, E-consult- electronic consult, ACE-I- angiotensin converting enzyme inhibitor; ARB- angiotensin receptor blocker, RRT- renal replacement therapy, PROMs- patient reported outcome measures

The CKD registry facilitates data driven population health interventions, whereas previous efforts have been based on national data trends. The goal is to slow the progression of renal disease, prevent complications, and optimize transitions along the CKD spectrum (Fig. 3) [7].Our organization is currently utilizing the CKD registry to deploy population health management strategies including e-consults, computer decision support (CDS) for nephrology referral, PROMs and ESRD care coordination. We are in the process of developing additional CDS for quality metrics, and implementing advanced CKD care coordination. The first challenge is to improve rates of CKD recognition. Our robust specialty e-consult program can facilitate early CKD management by PCPs with nephrology support, without in-person visits. Timely referral of CKD patients to nephrology can be supported via identification of patients at highest risk for progression and CDS. As patients progress through stages of CKD, renal replacement therapy (RRT) planning becomes as important as managing CKD complications. The registry can facilitate coordinated, multi-disciplinary care for advanced CKD by capturing timely, accurate data regarding progression, and the need for vascular access. Ideal application of a CKD registry involves population health coordinators who track performance data over time, and facilitate key care processes. The registry can be leveraged by coordinators to facilitate RRT initiation discussions, modality decisions and conservative or palliative care. Finally, we have incorporated PROMs data in our registry, to drive shared decision-making and gauge patients’ perception of their health. We chose the KDOQL-SF 1.3 to capture PROMS data, because it includes 43 kidney-disease targeted items and has been validated in chronic kidney disease across ethnic groups [24].

### Strengths

Our CKD registry has a number of strengths. First, it aggregates data from a large patient population in an integrated network. Second, our registry uses the EHR as a dynamic data source leveraging labs, medication/prescription data, billing, and clinician encounters. Third, our algorithms for CKD stage identification, vascular access, and transplant status were developed iteratively over 2 years based on feedback from PCP, nephrology and population health leadership. Fourth, we have included a broad range of metrics from early to advanced CKD, enabling use by both PCPs and nephrologists. Fifth, the tool is accessible both by individual providers and clinical directors engaged in clinical management as well as population health specialists interested in systems-based interventions. Finally, the registry incorporates important clinical outcomes like CKD progression, ED visits, and hospitalizations that can be utilized to evaluate the impact of interventions on outcomes.

### Limitations

Though our CKD registry has undergone multiple iterations before reaching its current state, it may benefit from further revision. Identification and stratification of patients continues to be a major challenge. Identification is inherently limited by screening, and efforts to promote screening are needed. Identification of patients with 5D CKD who receive dialysis at outside dialysis units, but receive inpatient and interventional nephrology care through our system, Our validation approach demonstrates that despite refinement of the 5D identification algorithm, there are limitations to labs, diagnosis codes and targeted word search. This is reflective of the dynamic status of CKD, but also exemplifies fragmented care, specifically RRT, that occurs in disparate locations.

Staging stratification based on eGFR is complex given the moving target in most patients. We define stage based on most recent eGFR for consistency, but the argument could be made for a blended average approach. With regards to loss to follow-up, patients without either a visit or a laboratory test within the past 5.5 years are dropped from the registry to focus care. Future iterations of the CKD registry may include follow-up with patients nearing the time for removal from the registry. Similarly, given that all Partners CKD patients are included in the registry, analysis is limited to positive predictive value and does not include sensitivity or specificity. Another limitation is that the registry is not currently linked to external registries such as USRDS or the Social Security Death Master File, which would ensure more accurate identification of 5D patients and mortality, respectively. Our utilization data is limited given that the registry is restricted to in-network visits, and therefore, may be underestimating total visits. Finally, the low rate of HBV vaccination among stage 5D patients may reflect the fact that they receive most of their care at dialysis units that do not share an EHR with PHS.

### Implications

We believe that institutions and networks should adopt EHR-based CKD registries as we move towards value-based care. There is a need for regional and institution-based registries as there is known regional variation in care delivered. Furthermore, as cardiovascular disease remains the greatest cause of mortality in CKD patients, the inclusion of data regarding cardiovascular risk factors in this registry will foster robust multi-disciplinary cardiovascular risk optimization.

## Conclusions

We have presented a large-scale, network-based electronic CKD registry that identifies key opportunities to improve care delivery across the spectrum of the condition. Additional study is needed to test the hypothesis that leveraging our CKD registry to implement population health strategies will positively impact the clinical, societal and economic burden of the disease.

## Additional files

Additional file 1: **Table S1.** Stage 5D Classification criteria in PHS CKD Registry. Variables considered by algorithm for inclusion into or exclusion from stage 5D. (DOCX 31 kb)

Additional file 2: **Figure S1.** Partners Healthcare System CKD Registry. Screenshot of registry view seen by clinicians accessing the registry for clinical care purposes. (DOCX 409 kb)

Additional file 3: **Table S2.** List of renally unsafe medications for patients with eGFR < 30 ml/min. List, developed by PHS CKD Collaborative, of renally unsafe and potentially unsafe medications that are reviewed by registry algorithm for each patient. (DOCX 30 kb)

## Abbreviations

ACE-Is: Angiotensin converting enzyme inhibitors
ARBs: Angiotensin receptor blockers
AVF: Arteriovenous fistula
AVG: Arteriovenous graft
BWH: Brigham and Women’s Hospital
CDS: Clinical decision support
CHF: Congestive Heart Failure
CI: Confidence interval
CKD: Chronic kidney disease
CKD-EPI: Chronic Kidney Disease Epidemiology Collaboration
COPD: Chronic Obstructive Pulmonary Disease
CPT: Current Procedural Terminology
CVD: Cardiovascular disease, including Coronary Artery Disease
E-consult: Electronic consult
ED: Emergency Department
eGFR: Estimated glomerular filtration rate
EHR: Electronic health record
HBsAb: Hepatitis B Surface Antibody
HBV immunization: Hepatitis B virus
HTN: Hypertension
MDRD equation: Modification of Diet in Renal Disease
MGH: Massachusetts General Hospital
PCPs: Primary Care Providers
PHS: Partners Healthcare System
PPV: Positive predictive value
PROMs: Patient reported outcome measures
PVD: Peripheral Vascular Disease and Stroke
RRT: Renal Replacement Therapy
urine MALB/creat: Urine albumin to creatinine ratio
urine prot/creat: Urine protein to creatinine ratio
USRDS: United States Renal Data System
