# Supplementary material for: Development of an electronic health record-based chronic kidney disease registry to promote population health management
Source: BMC Nephrol. 2019 Mar 1;20:72. doi: 10.1186/s12882-019-1260-y (PMC6397481; doi:10.1186/s12882-019-1260-y)
Supplement: Supplementary file 1 — Table S1. Stage 5D Classification criteria in PHS CKD Registry. Variables considered by algorithm for inclusion into or exclusion from stage 5D. (DOCX 31 kb) [file 12882_2019_1260_MOESM1_ESM.docx]

Additional file 1: **Table S1** Stage 5D classification criteria in PHS CKD registry

| **Variables considered inclusion algorithm** |  |
| --- | --- |
|  | active problem list diagnosis for dialysis and ESRD |
|  | visit diagnoses for dialysis and ESRD |
|  | billing diagnoses for dialysis and ESRD |
|  | outpatient hemodialysis or CAPD orders |
|  | dialysis nephrology progress note |
|  | peritoneal dialysis listed in dialysis history without end date |
|  | dialysis AV access |
|  | CPT codes for outpatient dialysis services |
| **Variables considered exclusion algorithm** |  |
|  | eGFR_1_ and eGFR_2_ ≥ 20 |
|  | eGFR_1_ date 30 days after kidney transplant and is eGFR_1_ ≥ 20 |

**Abbreviations:**

eGFR- estimated glomerular filtration rate

ESRD- End Stage Renal Disease

**Definitions:**

eGFR_1_=The most recent eGFR value within the last 3 years

eGFR_2_=The second most recent eGFR value with the last 3 years

Billing data available within electronic health record which includes diagnoses of ESRD or dialysis

CPT codes = 9018-25, 90963-70, G0309, G9013-4, 90935, 90937, 90945, 90947, 99512, 0505F, 4052F, S9335

Diagnosis codes= ICD-10 codes (i.e. E 10.22, E11.22 etc.)
