# Supplementary figures and images for: Development of an electronic health record-based chronic kidney disease registry to promote population health management
Source: BMC Nephrol. 2019 Mar 1;20:72. doi: 10.1186/s12882-019-1260-y (PMC6397481; doi:10.1186/s12882-019-1260-y)

Additional file 2: **Figure S1** Partners Healthcare System CKD Registry


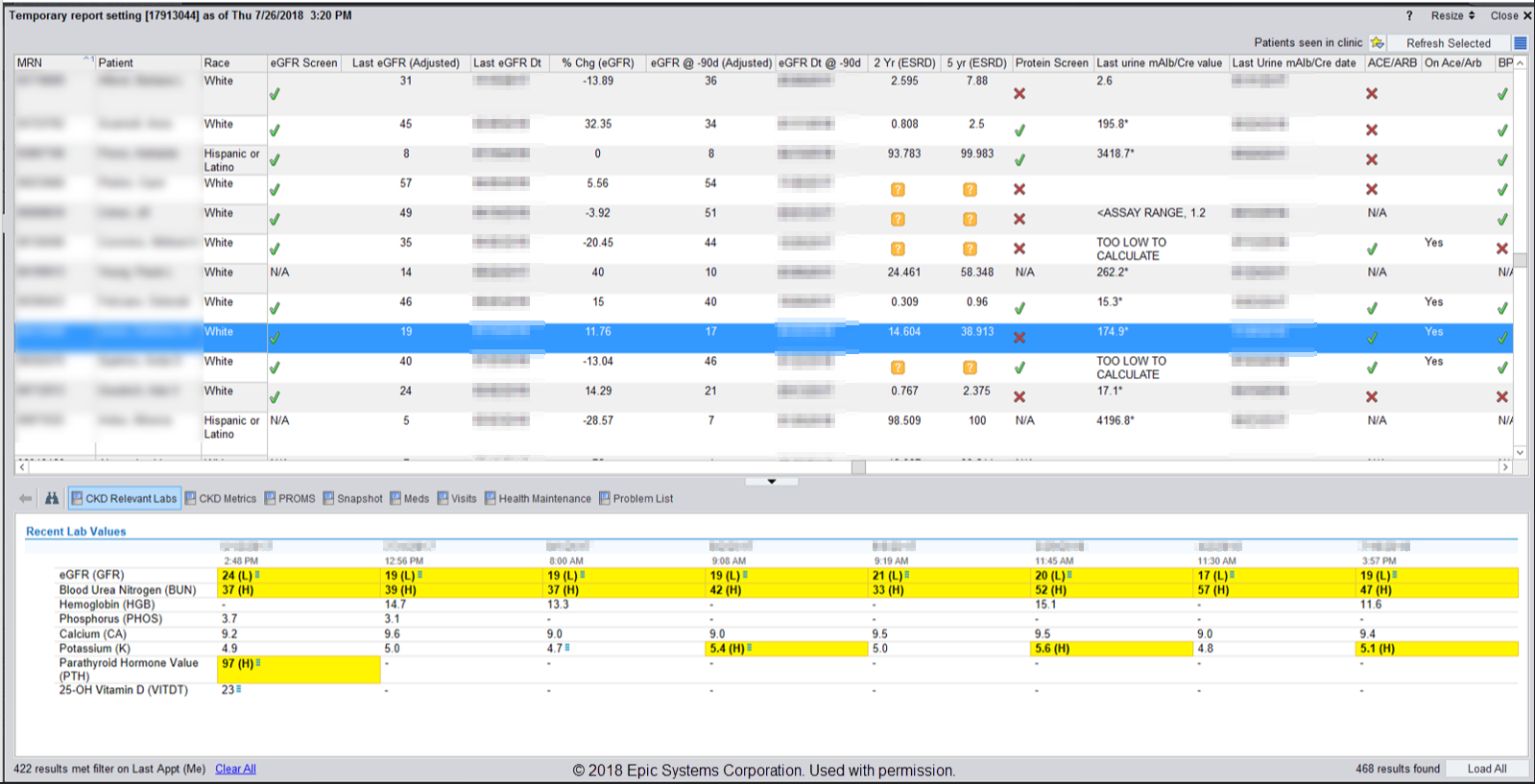

Supplement: Supplementary file 2 — Figure S1. Partners Healthcare System CKD Registry. Screenshot of registry view seen by clinicians accessing the registry for clinical care purposes. (DOCX 409 kb) [file 12882_2019_1260_MOESM2_ESM.docx]
