# Supplementary material for: Development of an electronic health record-based chronic kidney disease registry to promote population health management
Source: BMC Nephrol. 2019 Mar 1;20:72. doi: 10.1186/s12882-019-1260-y (PMC6397481; doi:10.1186/s12882-019-1260-y)
Supplement: Supplementary file 3 — Table S2. List of renally unsafe medications for patients with eGFR < 30 ml/min. List, developed by PHS CKD Collaborative, of renally unsafe and potentially unsafe medications that are reviewed by registry algorithm for each patient. (DOCX 30 kb) [file 12882_2019_1260_MOESM3_ESM.docx]

Additional file 3: **Table S2** List of renally unsafe medications for patients with eGFR < 30 ml/min

“**Renally unsafe medications**”

1. Metformin HCL
2. Alendronate Sodium
3. Risedronate Sodium
4. Ibandronate Sodium
5. Zoledronic Acid
6. Nitrofurantoin
7. Rivaroxaban
8. Metformin/AA 7/Herb 125/ Choline
9. Metformin/CAFF/AA7/HRB125/Chol
10. Canagliflozin/metformin HCL
11. Empagliflozin/metformin HCL
12. Glipizide/metforminHCL
13. Glyburide/metformin HCL
14. Linaglipin/metformin HCL
15. Repaglinide/metformin HCL
16. Rosiglitazone /metformin HCL
17. Alogliptin Benz/metformin HCL
18. Pioglitazone HCL /metformin HCL
19. Sitagliptin Phos/metformin HCL
20. Saxagliptin HCL/metformin HCL
21. Nitrofurantoin Macrocrystal
22. Nitrofurantoin Monohydrate/M-Cryst
23. Risedronate Sodium/Calcium Carb
24. Zoledcronic Acid/Mannitol-Water
25. Alendronate Sodium /Vitamin D3

“**Potentially unsafe medications**”

1. Digoxin
2. Levofloxacin
3. Atenolol
4. Lithium
5. Neurontin (Gabapentin)
6. Bactrim (trimethoprim-sulfomethoxazole)
